# Long-term antibacterial performances of biodegradable polylactic acid materials with direct absorption of antibiotic agents†

**DOI:** 10.1039/c8ra00504d

**Published:** 2018-05-01

**Authors:** Chien-Hao Chen, Yuan-Yuan Yao, Hao-Che Tang, Tung-Yi Lin, Dave W. Chen, Kong-Wei Cheng

**Affiliations:** Department of Orthopaedic Surgery, Chang Gung Memorial Hospital Keelung Branch Taiwan mr5181@adm.cgmh.org.tw +886-3-3278113 +886-3-3281200-2420; College of Medicine, Chang Gung University Taoyuan Taiwan; Department of Chemical and Materials Engineering, Chang Gung University 259 Wen-Hwa 1st Rd.,Kwei-Shan, 333 Taoyuan Taiwan kwcheng@mail.cgu.edu.tw +886-3-2118668 +886-3-2118800-3353

## Abstract

In this study, polylactic acid (PLA) disks with antibacterial performances were prepared using 3D printing technology combined with direct adsorption of the antibiotic agents in solution baths. The effects of the layer thicknesses for the building of the 3D printing PLA disks and the amounts of antibiotic agents absorbed onto the sample surfaces on their antibacterial activities were investigated. The antibiotic agent release profiles from the samples surface into the buffer solution show that the antibacterial performances of these samples can reach up to 28 days. With a decrease in the concentration of antibiotic agent in the solution bath, the amount of antibiotic agent adsorbed on the sample surfaces also decreases, but their antibacterial performances can still maintain at least 7 days. In the bioactivity tests of the various organisms, the release amount of antibiotic agent from the sample can inhibit *E. coli* and *S. aureus* for over 80% up to 28 days. In the antibacterial activity tests, a PLA disk with suitable antibiotic agents covering its surface has a good inhibitory effect on the growth ability of *S. aureus* of less than 50% in six hours.

## Abstract 2

In this study, we developed a surface modification of 3D printing PLA disks. The relative optical density of the *S. aureus* in the solution can reduce to 40% using the PLA disk directly absorbed with suitable antibiotic agents.

## Graphical abstract

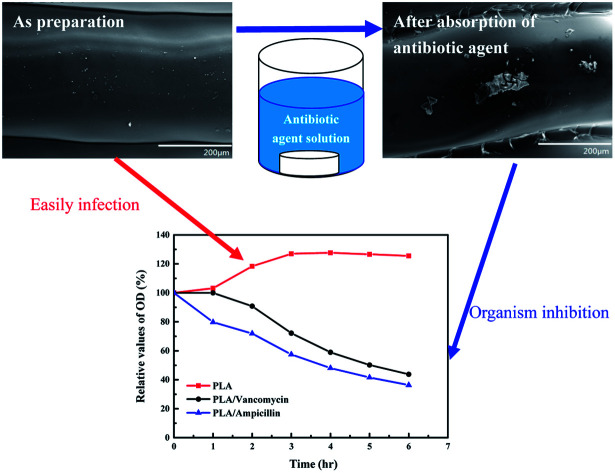

## Introduction

Biodegradable polymers such as polylactic acid (PLA) have been widely used in the development of tissue engineering, biodegradable implants, sutures, and the control of drug release rate.^1–3^ PLA is also a low crystalline polymer used as the organic precursor for 3D printing technology. The 3D printing technology using fused deposition with the low crystalline/amorphous polymers such as PLA, polyacylonitrile/butadiene/styrene or polyester as the precursors, the digital light projection technology with photo-sensitive resins, or the selective laser sintering technology with the semi-crystalline polymers have been developed for the applications of drug products, industrial molds and possible applications of biodegradable bone-supports.^4–10^ Using a suitable design for the products, a 3D object with the layer-by-layer building by using the 3D printer can be obtained. Norman *et al.* (2017)^5^ reviewed the benefits and the methodology of 3D printing in the pharmaceutical and biological manufacturing applications. The applications of 3D printing have many benefits such as the un-moldable printing, infinite variety of shapes for printing, rapid printing from the digital design and printing at the point-of-care. An important benefit in the treatment of complex musculoskeletal wound for the orthopedic sufferer is the rapid printing and infinite variety of shapes for the printing. Therefore, we can design the implant supports with complex shapes for the orthopedic sufferers and decrease manufacturing time compared with the traditional heterogeneous implants production process. Although we can solve the problem about the long manufacturing time for these heterogeneous implants, the antibacterial property of these implants using for the complex musculoskeletal wounds is still the major problem and influences the recovery time of the sufferers. Maintaining the long-term antibacterial activities for these heterogeneous implants is the important key factor for postoperative cares of the sufferers. The possible methods for the development or modification of the surfaces for these heterogeneous implants with suitable antibacterial properties thus become the important research fields. Hassan *et al.* (2013)^11^ reviewed the possible ways for the surface modifications of these biomaterials with the certain antibacterial properties. Current approaches for the surface modifications of biomaterials include the surface coating with silver or titanium ions, the surface polymerisation of the antimicrobial agents, and introduction of the functional groups on the sample surface using polymerisation or plasma treatments. However, coating with the transitional metal ions on the heterogeneous implant surfaces may have some uncertain dangers in the treatment of orthopedic surgery. Another possible choice is the coating with the biodegradable polymer thin film containing the antibiotic agent on the heterogeneous implant surfaces in order to prevent infections of organisms. The biodegradable polymer/antibiotic agent composites can provide the local antibiotic delivery way in the musculoskeletal wounds. A biodegradable carrier such as the poly-lactic-*co*-glycolic acid (PLGA) containing the antibiotic agent with appropriate concentration, which can provide the stable drug release rate, is thus used as attractive treatment option for minimizing the infections in complex musculoskeletal wounds.^12,13^ Although the above treatments are the main methods for minimizing infections in musculoskeletal wounds, these surface modifications of the heterogeneous implants with the stable and long-term drug release rate are not enough to meet the clinical requirements. Even with the advance in surgical technique and the availability of newly developed antibiotic, infection of the sufferers is still the problem in the management of open fractures related diseases. The high cost of biodegradable PLGA is still another problem for the large-scale application in the surface modification of these heterogeneous implants. The poor material antibacterial properties and high costs for these heterogeneous implants, which may result in postoperative infection, make the waste of medical resources and time for the treatments of the orthopedic sufferers. The improvements of antibacterial properties of these heterogeneous implants, which has to meet with the clinical requirements, are the important objective of this study. We combined the medicine and material concepts to prepare the heterogeneous implants and developed the possible surface modifications for these heterogeneous implants in order to meet the clinical requirements. The 3D printing technology was employed for the preparation of heterogeneous implants and the development of the possible way for the surface modification of the samples was also carried out in order to increase their antibacterial properties. The *in vitro* drug release profiles of the antibiotic agents from the surfaces of PLA disks into the buffer solutions were analysed using the high performance liquid chromatography (HPLC). The bacterial inhibition tests and optical densities of the organisms in the Nutrient Broth or Miller's Broth solutions were also carried out in this study to evaluate the antibacterial properties of the PLA disks with and without the surface modifications.

## Experiments

In this study, we examined the possible way for the surface modification of PLA disks with coating the biodegradable polymer and/or antibiotic agents in order to enhance their anti-bacterial properties. The antibiotic agents of ampicillin sodium salt (C_16_H_18_N_3_NaO_4_S, purity > 98%) and the vancomycin hydrochloride (C_66_H_75_Cl_2_N_9_O_24_ HCl, purity > 98%) were provided from the Aldrich Co. Biodegradable PLGA (weight percent ratio of lactide : glycolide = 50 : 50, purity > 98%) and the phosphate buffer saline (PBS) solution with the purity of greater than 99.9% were provided from the Aldrich Co. The organisms used for the bacterial inhibition tests are the *Escherichia coli* (*E. coli*, FYE 678, DH5α) and the *Staphylococcus aureus* (*S. aureus*, ATCC6538R) provided from the Bioresource Collection and Research Center (BCRC, Taiwan). The Miller's LB Broth (LB, tryptone 10 g, yease 5 g and NaCl 10%) and Nutrient Broth (NB, beef extract 3%, peptone 5 g) were used for the tests of bioactivity of *E. coli* and *S. aureus*, respectively. The PLA wires with the diameter of 1.75 mm for the 3D printing are provided from the Black Magic 3D Co. (USA). For the tests of antibacterial properties of PLA implants, a 3D printer (Prusa i3, Black Magic 3D Co. USA) with the fused-deposition modelling was employed to prepare the PLA disk for the simulation of heterogeneous implant. The PLA disk with the diameter of 2.5 cm and the thickness of 0.3 mm was produced using the 3D printer with the PLA layer thickness of 0.1–0.3 mm and printing speed of 25 mm s^−1^. The surface morphology, thermal properties and the surface area of the PLA disk were examined using the scanning electron microscope (S-3000N, Hitachi) with the acceleration voltage of 15 kV and working distance of 15 mm, thermogravimetric analyser (TGA, TATGAQ 50), differential scanning calorimeter (DSC, TADSC 50) and specific surface area micromeritics (ASAP 2020).

The surface modifications of the PLA disks were carried out using the coating PLGA/antibiotic agents and the directly absorption of antibiotic agents onto the PLA disk. For the coating the PLGA/antibiotic agents onto the PLA disk, a precursors solution with the weight percentage of 12.5% for ampicillin and 87.5% for PLGA were first dissolved in an acetone solution (purity > 99%) with the magnetic stirring, which was used as the coating parameter by Chen *et al.* (2012).^12^ The PLGA/antibiotic agent solution was coated onto the surface of PLA disk using the dip coating. This coating process was repeated at least five times in order to make the surface of PLA disk covering with the PLGA/antibiotic agent. Another approach for the surface modification was the direct absorption of antibiotic agent on the surface of the PLA disk. Various concentrations of antibiotic agents in the water baths were used for the preparation of PLA disk with the antibacterial properties. The 3D printing PLA disks were direct placed in the aqueous solution containing various concentrations of antibiotic agents at the room temperature. After the full absorption of antibiotic agents on the sample surface, the PLA disks were kept in a clean container in order to avoid any possible influence from other chemicals or organisms.

The *in vitro* elution test was carried out for the determination of drug release rate from the PLA disk with the surface modifications. Similar approaches have been reported in the literature.^14,15^ We prepared the phosphate buffer solution (pH 7.4, 25 mL) as the dissolution medium for the tests of drug release behaviour from the PLA disk. The PLA disks with surface modification were incubated in the phosphate buffer saline at the temperature of 37 °C with the wavering rate of 30 rpm. The phosphate buffer medium for the *in vitro* elution test was analysed with the time interval of 24 h using the HPLC (Pu-2080, JASCO Co.) with the SYMMETRY C_8_ column (4.6 × 250 mm, Shim-pack, VP-ODS). The phosphate buffer solution (25 mL) was replaced every 24 h in order to avoid the influence of saturated concentration of antibiotic agent in the buffer solution. The mobile phase for the analysis of concentration of antibiotic agent in the buffer solution was the mixture of acetonitrile and water (35% : 65% v/v). The absorbency was kept at 220 nm and the flow rate of mobile phase was kept at 1 mL min^−1^ for the analysis of amount of antibiotic agent in the buffer solution. The calibration curves for the antibiotic agents in the water bath and phosphate buffer solution (both correction coefficient > 0.99) were made for the determination of unknown concentrations of the antibiotic agents in the aqueous or buffer solutions.

The test of bioactivity for the released antibiotic agent on the organism has been discussed in the literatures.^12,15,16^ The method for the test in the study is the same with that reported by Chen *et al.* (2012).^12^ Here shows the brief description. 200 μl samples with *E. coli* or *S. aureus* inoculum were cultured in 5 mL Miller's LB Broth and Nutrient Borth, respectively, and grown in 12 hours at 37 °C with the constant shaking rate of 200 rpm. Finally, the concentration of bacterial suspensions was adjusted of around 10^8^ colony-forming unit (CFU)/mL. The antibiotic disk diffusion method for the *E. coli* in agar containing LB and *S. aureus* in the agar containing NB was carried out in Petri dishes, respectively, in order to evaluation the antibacterial properties of the PLA disk with the absorption of antibiotic agents. 200 μl of solution containing organisms with the bacterial concentration of 10^8^ CFU mL^−1^ were pipetted and seeded onto the agars in the Petri disks for the tests. The inhibition zones were measured after 16–18 h of incubation at 35 °C. A calibration curve for the inhibition of each organism was also determined by the standard concentrations of antibiotic agent (1, 10, 100 and 1000 μg mL^−1^). The release concentration of antibiotic agents was then determined by interpreting the curve. The bioactivity of the antibiotic agent on organism (*E. coli* and *S. aureus*) was calculated using the following equation:1
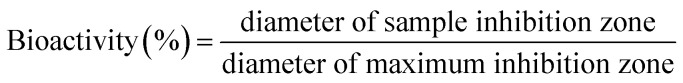


For the test of optical density of the bacterial suspension solution with and without the antibiotic agent absorbed on the surfaces of PLA substrates, two concentrations of bacterial suspension solutions (10^6^ CFU mL^−1^ and 10^8^ CFU mL^−1^) for the *S. aureus* were employed in order to estimate the antibacterial activity of the PLA substrate with surface modification. The PLA substrate was kept at the antibiotic agent solution (3 mL) with the concentration of 50 mg mL^−1^ for the ampicillin sodium salt and the vancomycin hydrochloride in five days, respectively. The standard test with only organism in the solution was also carried out in order to estimate the growth rate of organism as a function of time. The bacterial suspension solutions were kept at 37 °C with the shaking rate of 180 rpm. The values of optical density for the bacterial suspension solutions with and without the antibiotic agent on the surfaces of PLA disk were measured using the scanning spectrophotometer (Shimadzu, UV-1601PC) with the incident light wavelength of 600 nm. The relative value of optical density of the bacterial suspension solution was calculated using the following equation:2
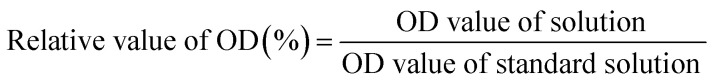


## Results and discussion

Because the shape or size for the biodegradable implants was depended on the requirements of the orthopedic sufferers, the short production time for the heterogeneous implant is thus necessary. The 3D printing technology is an interesting and fast production technology that can easily prepare the implant for the sufferer. However, the antibacterial property of these heterogeneous implants is the major factor that influences the recovery time of the sufferers. The development of the possible surface modification way with the low-cost, easily preparation and long antibacterial properties of these implants is the major target of this study. In order to examine the possible way for the surface modification of these implants, the simple and symmetrical disk was prepared using the 3D printing with the PLA as the material precursor. A 3D printer using the fused deposition modelling technology was employed to prepare the PLA disk. In order to set the nozzle temperature for the 3D printer, the thermal properties of PLA precursors were first examined using the TGA and DSC. Fig. S1(I) and (II)† show the thermal properties of PLA precursors using the DSC and TGA analysis. From the result shown in Fig. S1(I),† the glass-transition temperature (*T*_g_), crystallization temperature (*T*_c_) and melting temperature (*T*_m_) of PLA wires are around 60 °C, 100 °C and 170 °C, respectively. The thermal properties of PLA precursors agree well with those reported by Xiao *et al.* (2012).^17^ Using the DSC curve shown in Fig. S1(I),† we can estimate the crystallinity of PLA precursor used in this study of around 9%, which indicates that the PLA precursor is the low crystallinity polymer. The results shown in Fig. S1(I)† indicate that the temperature of nozzle for the 3D printer has to be set of higher than 170 °C. Then we checked the traditional TGA curves of PLA shown in Fig. S1(II).† It can be found that the thermal degradation temperature of around 350 °C for the PLA sample. From the results of TGA and DSC curves of PLA sample, the temperature of nozzle for the 3D printer has to be set in the range of 170–350 °C. After we tested various possible temperatures set at the nozzle for 3D printer, the temperature of nozzle of around 220 °C for our 3D printer is good parameter for the production of the PLA disks. The 3D printer used in this study is the fused deposition method, which was design by using the heating at the nozzle that distributed extruded polymer as a fine filament in the layer-by-layer building approach.^4^ The layer thickness for the building of our PLA carriers is also the parameter that may influence the antibacterial property of PLA sample. In this study, we set three possible layer thicknesses for the building of PLA samples by using the 3D printer. Fig. S1(III)† shows the SEM images of PLA disks with the layer thickness of 0.1 mm (left) and 0.3 mm (right). The insert figure is the picture of PLA disk prepared using the 3D printer. Total layer with the layer thickness of 0.1 mm for PLA disk is higher than that with the layer thickness of 0.3 mm for PLA disk. Different layer thickness for the building of the PLA disk may result in different surface area, which may influence the drug release rate of antibiotic agent loading at the PLA disk surfaces.

Because the surface area of the PLA disk is important for the loading of antibiotic agent, we used specific surface area micromeritics to estimate its surface area. The efficient surface area for the samples with the layer thickness of 0.1, 0.2 and 0.3 mm for the building of our PLA disks are around 0.16, 1.8, 3.9 m^2^ g^−1^, which increase with an increase in the layer thickness set for the 3D printing. The large efficient surface area of sample may have high antibiotic agents loading, which have long-term antibacterial performances. Because the sample with the layer thickness of 0.3 mm has largest surface area, it was used to analyse possible surface modification way that has the good antibacterial property. First, a PLA disk was coated with the PLGA/ampicillin thin film using the directly dipping coating. Fig. S2(I)† shows the release characteristics of ampicillin from the PLA disk with the coating of PLGA/ampicillin thin-film in the buffer solution. The values of minimum inhibition concentration (MIC) of 90% for *E. coli* (dish line) and MIC 90 and 50% for *S. aureus* (black line) obtained from the literatures are also shown in Fig. S2(I).†^18,19^ The release profile of ampicillin from the PLGA/ampicillin thin film on the PLA disk can continue around 22 days. However, the concentrations of ampicillin in the buffer solutions at the 10–12 days were lower than the value of MIC 90 for *E. coli* but the concentrations of ampicillin in the buffer solutions were still higher than the value of MIC 90 for *S. aureus* during the test. The concentration of ampicillin in the buffer solution at the first eight days were in the range of 200–1000 μg mL^−1^. The concentration of ampicillin in the buffer solution decreased very fast in the 9^th^ to 11^th^ days. The possible reason for the release profiles of ampicillin from the samples in the first 8 days may be due to the drug released from the surface of PLGA/ampicillin thin film on the PLA disk. The concentration of ampicillin in the buffer solution dropped very fast in 9^th^ to 11^th^ days due to the exhausted of the ampicillin at the surface of PLGA/ampicillin thin films. We can observe that the concentration of ampicillin in the buffer solution increase at 11^th^ day due to the biodegradation of PLGA thin film occurred and make the release of ampicillin from the inside of PLGA/ampicillin thin film to the buffer solution. Because the concentration of ampicillin in the buffer solution from the PLGA/ampicillin thin film are not stable in the whole test and the high cost of PLGA, we designed another possible approach for the surface modification of the PLA disk. Since the PLA is also the polymer with many hydroxyl function groups, which can form the chemical bonding with the vancomycin and ampicillin. The directly absorption of antibiotic agent in the surface of PLA disk is possible because of the interaction between the PLA disk and the antibiotic agent. Then we put the PLA disk in the 3 mL aqueous solution containing the ampicillin with the concentration of 50 mg mL^−1^. The absorption time of ampicillin on the surface of PLA disk was kept at 1 hour for this pre-study. The release profile of ampicillin from the PLA disk with the directly absorption of the antibiotic agent is also shown in Fig. S2(I).† The results showed that the stable release profile of ampicillin from the sample into the buffer solution could continue around of 22 days. Fig. S2(II)† also shows the release profile of ampicillin from the PLA wires with the same experimental test. Very low absorption amount of ampicillin could be observed on the surface of the PLA wires before 3D printing. Compared with the release profile of ampicillin in the buffer solution from the biodegradation of PLGA on the PLA disk, the directly absorption of antibiotic agent on the PLA substrate for the maintaining its antibacterial property is the low-cost and simple process for the preparation of PLA substrates with good antibacterial properties.

Then we designed several approaches to examine the possibility for the directly absorption of antibiotic agent on the PLA disks. The antibiotic agents for these tests were the ampicillin and vancomycin. Because the surface areas of the PLA disk with various layer thicknesses show different values, the influence of layer thickness may result in the different drug release profile in buffer solution. First, an aqueous solution with the total volume of 3 mL and the concentration of 50 mg mL^−1^ of ampicillin was used to examine the release profiles of antibiotic agents from the PLA disks with various layer thicknesses prepared using the 3D printing. In order to make all ampicillin in the aqueous solution absorbed onto the surface of PLA disk, the absorption time for PLA disk in the aqueous solution containing ampicillin was increased from 1 hour to five days. After the absorption process, the concentration of residual ampicillin in the aqueous solution was analysed by using the HPLC measurement. Table 1 shows the absorption percentages of ampicillin on the surface of PLA disks. Samples (A)–(C) corresponded to the layer thickness of 0.1, 0.2 and 0.3 mm by using the fused deposition modelling 3D printer. All absorption percentages of ampicillin on the sample surfaces are higher than 90%. The loading amount of ampicillin on the samples (A)–(C) were 74.8, 73.1 and 72.6 mg-ampicillin/g-PLA disk. The results reported in the Table 1 indicated no apparent influence of layer thickness for the PLA disk with the absorption amount of ampicillin on the PLA disk or the same active area for absorption of ampicillin on the PLA disk although the values of surface areas of various PLA disks are different.

**Table 1:** Test parameters for the drug release with various antibiotic agents used in this study

Sample	Layer thickness/antibiotic agents	Concentration of antibiotic agent for absorption	Absorption percentage (%)	Rate constant for K–P model (d^−1^)	*n* values for the K–P model	Value of *R*^2^
(A)	0.1 mm/ampicillin	50 mg mL^−1^	94.9	4.56	0.51	0.99
(B)	0.2 mm/ampicillin	50 mg mL^−1^	91.8	5.23	0.48	0.99
(C)	0.3 mm/ampicillin	50 mg mL^−1^	90.1	5.53	0.51	0.98
(D)	0.3 mm/vancomycin	10 mg mL^−1^	91.5	22.38	0.99	0.99
(E)	0.3 mm/vancomycin	30 mg mL^−1^	86.8	10.26	0.94	0.99
(F)	0.3 mm/vancomycin	50 mg mL^−1^	82.5	7.71	0.75	0.98

Because the measurement method for the sample surface area is based on the physical absorption, the absorption behaviour of the chemical absorption may be different with the results obtained from specific surface area micromeritics. Then we examined the ampicillin release profiles for samples (A)–(C) in the buffer solutions as a function of time in order to verify our assumptions.

**Fig. 1:**
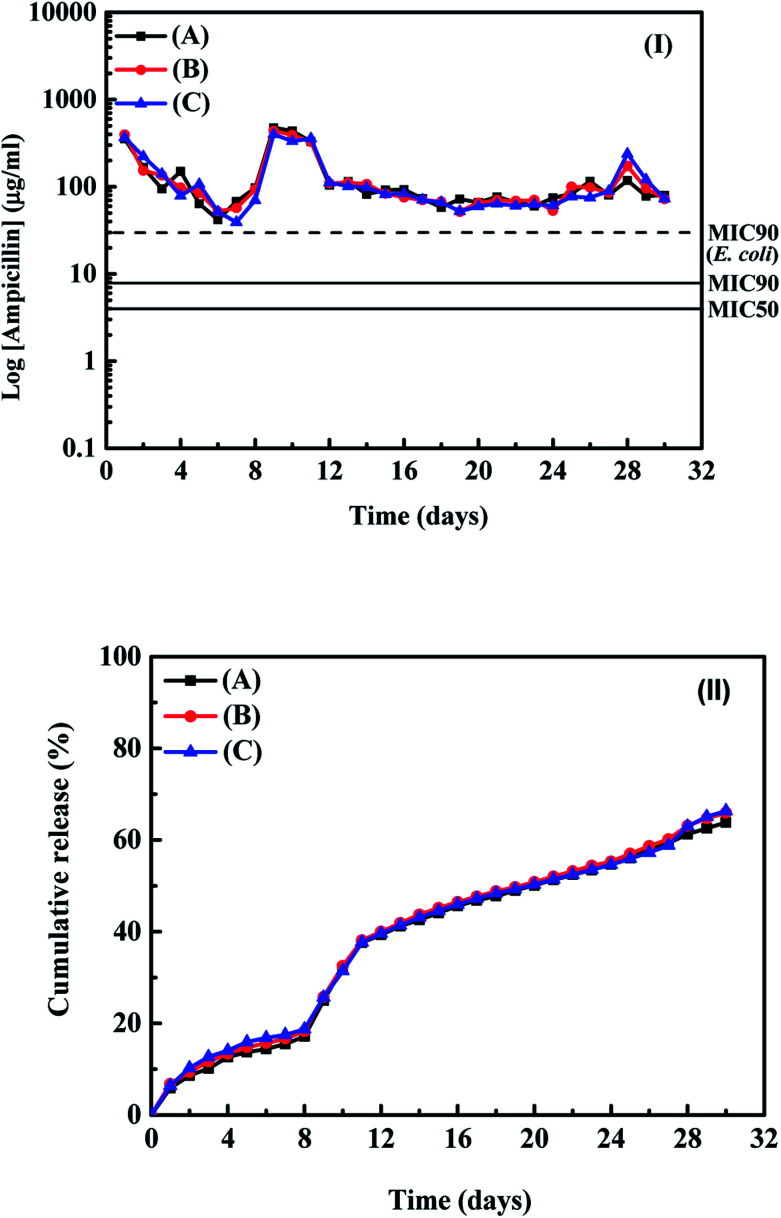
(I) Release profiles and (II) the cumulative amounts of ampicillin released from samples (A)–(C) into the buffer solution as a function of time.

Fig. 1(I) and (II) show the release profiles and cumulative amounts of ampicillin from the samples (A)–(C) in the buffer solution as a function of time, respectively. From the results in Fig. 1(I) and (II), the release time for the ampicillin from the samples into the buffer solution can continue at least 30 days and the concentrations of ampicillin in the buffer solutions for all samples are higher than values of MIC 90 for *E. coli* and *S. aureus*. It indicates that the ampicillin can release from the PLA disk and maintain the effective antibacterial properties with the directly absorption of antibiotic agents on the PLA disks. From the results shown in Fig. 1(I) and (II), we found three periods for the ampicillin release profiles from the PLA disks during the tests. For the first 7 days, the concentration of ampicillin released from the PLA disk decreased as a function of time and approached to the value of MIC 90 for the *E. coli*. The decrease of release concentration of ampicillin in the buffer solution was due to the physical desorption of ampicillin on the sample surface. A peak for the concentration of ampicillin in the buffer solution can be observed at the 8^th^ to 12^th^ days. According to the results reported by the Chen *et al.* (2012),^12^ Inzana *et al.* (2015)^20^ and Kim *et al.* (2017),^21^ the possible reason is due to the chemical desorption of ampicillin on the PLA disks or the degradation of polymer, which resulted in the peaks observed in Fig. 1(I) and (II). From the 12^th^ to 28^th^ days, a stable concentration of ampicillin in the buffer solution can be observed. From the results shown in Fig. 1(I) and (II), around 70% of ampicillin absorbed on the sample surface can be released into the buffer solution during around 30 days tests and maintain the concentration of antibiotic agent in the buffer solution of higher than the values of MIC 90 for *E. coli*, and *S. aureus*.

**Fig. 2:**
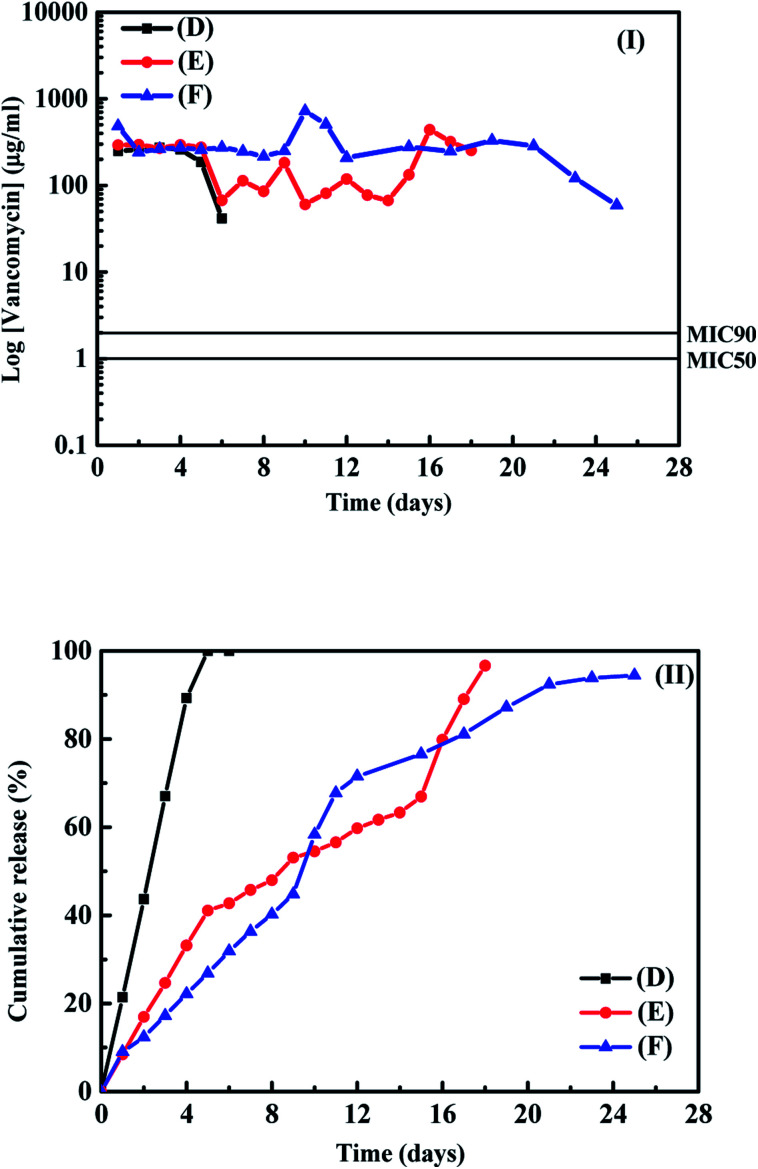
(I) The release profiles and (II) cumulative amounts of vancomycin from samples in the buffer solution as a function of time.

The results shown in Fig. 1(I) and (II) indicated that directly absorption of antibiotic agent onto the PLA disk is the low-cost method for the further application with the samples having suitable antibacterial properties. In order to check surface behaviour of PLA disk in the buffer solution, the surface morphologies for the as–preparation, after absorption of drug and after the drug release test for sample (C) were examined using the SEM. Fig. S3(I)–(III)† show the SEM images of samples for the as-preparation, after absorption of ampicillin and after the drug release test, respectively. The clean PLA layer surface for the as-prepared PLA disk and some ampicillin attached on the PLA surface after the absorption process can be observed in the SEM images, respectively. After the drug release test, the boundary of PLA layer became unclear and no apparent ampicillin absorbed on the sample surface. The SEM image shown in Fig. S3(III)† indicates that the degradation of PLA disk occurred during the test and make the ampicillin absorbed on sample surface release into the buffer solution. Because the values of surface area of the PLA disks showed no apparent influence on the absorption of ampicillin at the sample surface, we used the layer thickness of 0.3 mm for the building of PLA disk and employed them for the tests of release profiles for vancomycin absorbed on the PLA disk. The concentrations of vancomycin with 10, 30 and 50 mg mL^−1^ in the 3 mL aqueous solutions were used for the loading of vancomycin on the surface of PLA disk in order to estimate the influence of amounts of vancomycin absorbed on the sample surface on their drug release curves. Table 1 also shows the detail parameters and results for the absorptions of vancomycin on the PLA disks. Around 82–92% vancomycin in the aqueous solution can be absorbed on the PLA disks with the layer thickness of 0.3 mm. Sample (D), (E) and (F) corresponded to the PLA disks maintained in the aqueous solutions with the concentrations of vancomycin for 10, 30 and 50 mg mL^−1^, respectively. The loading amounts of vancomycin in the samples (D)–(F) were 14.8, 42.0, 66.5 mg-vancomycin/g-PLA disk, respectively. From the results reported in the Table 1, the amount of ampicillin loading on the PLA disk is a little higher than that of vancomycin, but the difference is not large. The amounts of antibiotic agents loading on the PLA disk are controlled by the concentrations of aqueous solution containing antibiotic agents. The drug release profiles and the cumulative amounts of vancomycin from the PLA disks for samples are shown in Fig. 2(I) and (II) respectively. For sample (D) in the buffer solution, the stable vancomycin concentration released from the PLA disk could be observed in the first 4 days and decreased very fast at the 5^th^ day. The possible reason for the short release time is due to low loading amount of vancomycin on the sample surface. Then we checked the cumulative amount of vancomycin from the sample shown in Fig. 2(II), almost 100% of vancomycin loading at the sample surface released into the buffer solution. The cumulative curve of vancomycin from the PLA disk is similar with the results reported by Murata *et al.* (2017)^22^ and Román *et al.* (2013).^1^ The behaviour of drug release profile for sample (D) is due to the vancomycin weakly absorbed on the sample surface. For sample (E), the tendency of drug release profile at first five days was almost the same with that of sample (D). The variation of drug release profile for sample (E) can be observed in the 6^th^ to 15^th^ days.

The drug release profile of vancomycin from the PLA disk in 6^th^ to 15^th^ days was influenced by the diffusion and the swelling of PLA disk in the buffer solutions.^1,23^ At the 16^th^ to 18^th^ days, a peak can be observed in the Fig. 2(I) for sample (E), it seems that the degradation of PLA at the sample surface take place and make all vancomycin absorbed on the sample surface release into the buffer solution. The result shown in Fig. 2(II) also indicated that all vancomycin absorbed at the surface of sample (E) run out at around of 18^th^ day test. For the sample (F), a stable drug release profile of vancomycin can be observed in the first ten days. A peak in Fig. 2(I) can be observed at the 10^th^ to 13^th^ days. The results for sample (F) is the same with that for ampicillin in the buffer solution and those reported in the literature.^12,20,21^ At around of 22^nd^ day, the concentration of vancomycin in the buffer solution decreased, which was due to the run out of antibiotic agent absorbed on the sample surface.^24,25^ The results shown in Fig. 2(II) indicated that the effective antibacterial properties of sample (D)–(F) are around 6, 18 and 26 days for the drug release tests. Because the release mechanism of antibiotic agent from the PLA disk is also important for the further application, we used four possible models to correct the results shown in Fig. 1 and 2. These four models are the zero order, first order, Higuchi and Korsmeyer–Peppas (K–P) models for the fitting our data shown in the Fig. 1 and 2. Detail discussion about these models can be obtained in the literature.^26–29^

**Fig. 3:**
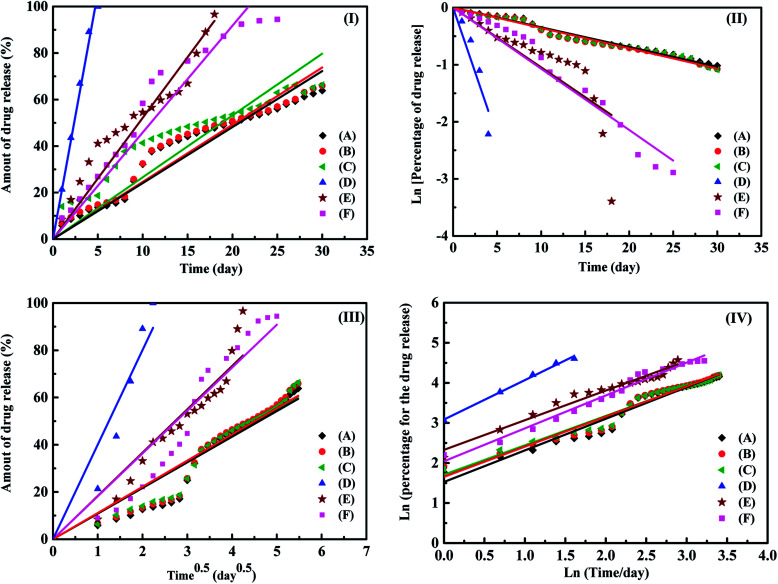
The fitting results of (I) zero-order, (II) first order, (III) Higuch and (IV) Koresmeyer–Pappas kinetic models for samples (A)–(F) in the buffer solutions.

**Fig. 4:**
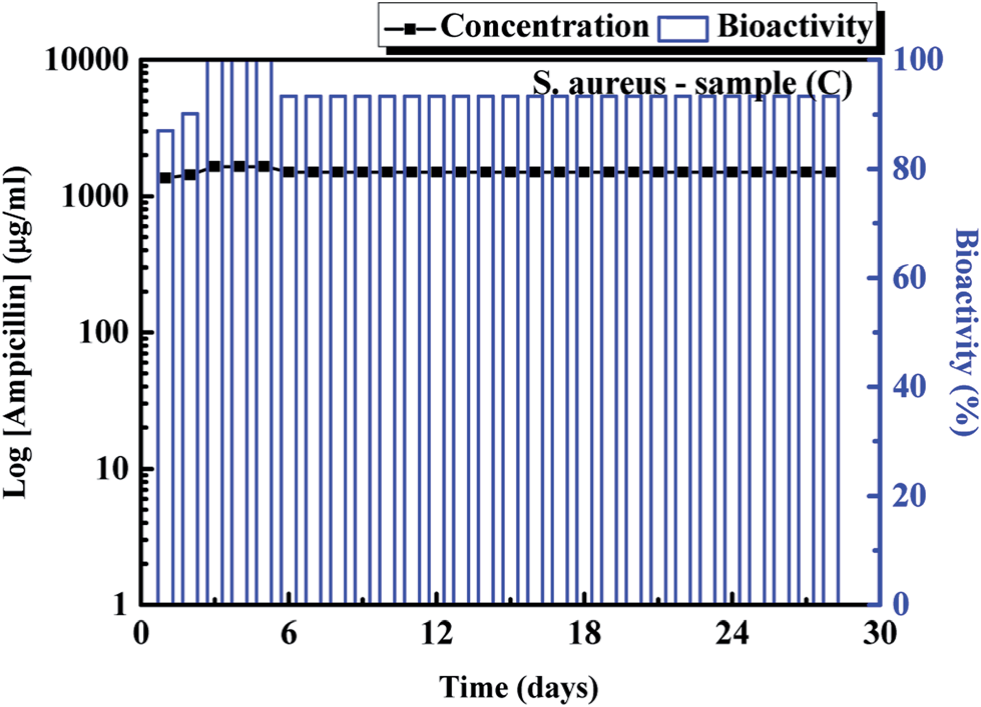
Bioactivity for *S. aureus* using the release of ampicillin from sample (C).

**Fig. 5:**
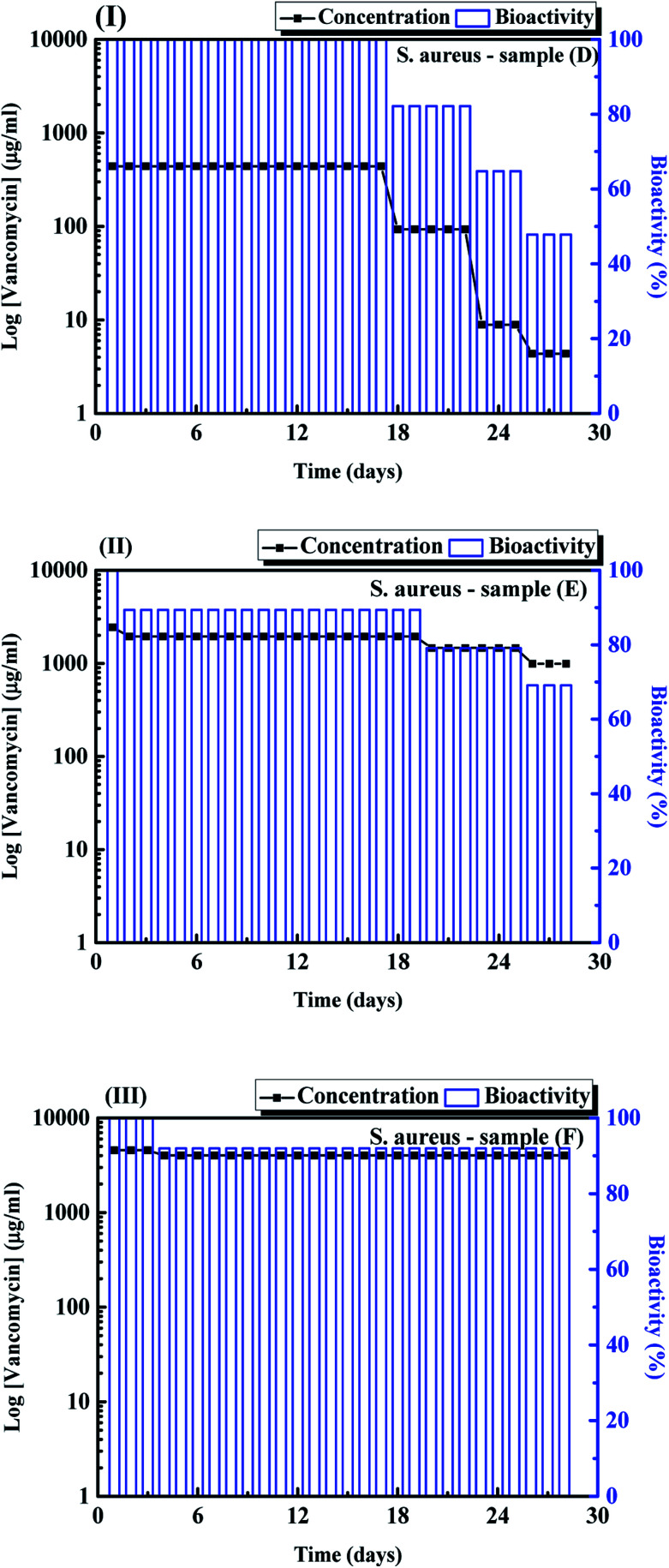
Bioactivity for *S. aureus* using the release vancomycin from samples (D)–(F).

Fig. 3(I)–(IV) shows the fitting results of samples (A)–(F) using the zero-order, first order, Higuchi and Korsmeyer–Peppas models, respectively. The results shown in Fig. 3 indicate that the Korsmeyer–Peppas model can give the good fitting results. The parameters of Korsmeyer–Peppas model for samples (A)–(F) are also shown in Table 1. The rate constants and *n* values for samples (A)–(C) are in the range of 4.5–5.3 day^−1^ and 0.48–0.51. The *n* values for samples (A)–(C) indicate that the drug release behaviour of ampicillin from the PLA disk is the Fickian model. The drug release profile of ampicillin from the PLA disk is controlled by the diffusivity of ampicillin in the buffer solution. For the samples (D)–(F), the rate constants for samples are in the range of 7.71–22.38 day^−1^. The rate constant for samples decreases with an increase in the vancomycin concentration in the aqueous solution. The highest rate constant for sample (D) indicates that the fast drug release rate because the antibiotic agents were just weakly absorbed on the sample surface. The *n* value for sample (D) indicated that the drug release mechanism approached to the zero-order release kinetic. For samples (E) and (F), the *n* values are in the range of 0.5–1.0, which indicates that the drug release profiles are controlled by diffusion and swelling of polymer. The swelling of polymer would result in the degradation of PLA disk due to the solvent transported into the polymer. For the treatments of musculoskeletal wound of the suffers, the influence of *S. aureus* is the major factor that influence the recovery time of the sufferers. Therefore, the bioactivity of *S. aureus* with samples (C) were then tested using the disk diffusion method. The result is shown in Fig. 4. For comparison, the bioactivity test of *E. coli* with sample (C) was also carried out using the same disk diffusion method. It is shown in Fig. S4.†Fig. 4 is the plot of bioactivities of *S. aureus* with sample (C) in the Petri disks as a function of time. The bioactivity of the antibiotics on the *E. coli* for sample (C) shown in Fig. S4† was in the range of 80–100%, while that of the *S. aureus* for sample (C) shown in Fig. 4 was higher than 90% in 24 days. The activity of ampicillin released from the sample (C) remained at least 24 days and maintained the high antibiotic activity on the *S. aureus*. Then we tested the bioactivity of *S. aureus* using the PLA disks with various amounts of vancomycin absorbed on the substrates (samples (D)–(F)). They are shown in Fig. 5(I)–(III), respectively. The bioactivity of the antibiotic agent for sample (D) on the *S. aureus* was kept at 100% and decreased at around 18^th^ day. After that, the bioactivity of antibiotics decreased due to the low concentration of vancomycin released from the sample (D). The bioactivity of antibiotics for sample (E) was maintained of higher than 90% in 20 days. At 20^th^ day, the bioactivity of antibiotics for sample (E) decreased but remained of around 70% in 28 days. The 90% bioactivity of antibiotics for sample (F) remained in the 28 days. It indicates that samples (C) and (F) have good and stable antibacterial properties in one month.

**Fig. 6:**
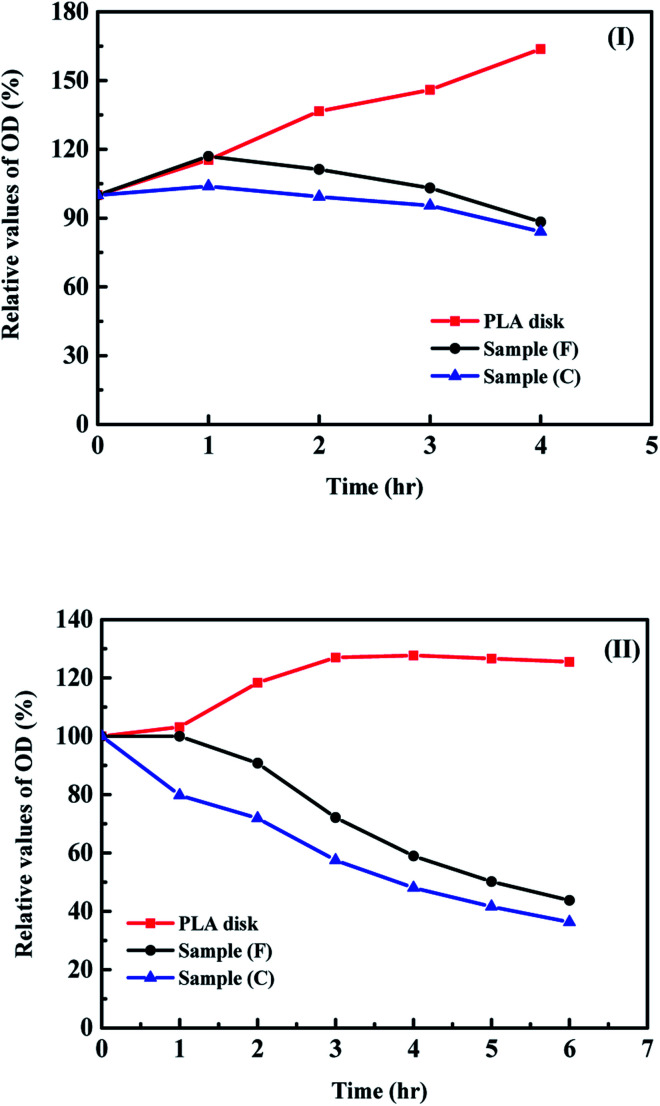
Relative optical densities values as a function of time for (I) the initial *S. aureus* concentration of 10^8^ CFU mL^−1^ and (II) 10^6^ CFU mL^−1^ with samples (C) and (F).

Because the *S. aureus* is the major organism that influences the recovery time of musculoskeletal wound of the sufferer. The measurements of optical density of the *S. aureus* in the solutions with and without antibiotic agents are important for the evaluation of behaviour of the bacterial growth.^30–32^ With an increase in the value of optical density for the solution containing organisms, the amounts of organisms in the solution increase while the decrease in the value of optical density, the growth of organisms in the solution are reduced. Fig. 6 shows the relative optical density (OD) values for the *S. aureus* in the solution containing the PLA disks with and without the surface modification of antibiotic agents. The OD values of *S. aureus* in the same solution without the PLA disk as a function of time was used as the standard test. For the high concentration of *S. aureu*s in the solution (Fig. 6(I)), the growth rate of *S. aureus* in the solution containing the PLA disk without any antibiotic agent coated on its surface increased very fast and was higher than the standard test. The results indicated only PLA disk without any surface modification make the fast growth rate of organisms on the substrates. It will make the possibility of infection for sufferers increase. With the surface modification of antibiotic agents on the PLA disk, the reduction of growth rate of organism in the solution can be observed and the antibiotic properties of sample (C) is better than that for sample (F). It may be due to the amounts of ampicillin loading on the PLA disk of higher than that for vancomycin, the different molecular weight or sensitivity to the *S. aureus* between ampicillin and vancomycin. At the lower concentration of organism in the solution (Fig. 6(II)), the relative OD values can be reduced to around 40% in 6 hours. These results show the directly absorption of antibiotic agents on the PLA disk have good antibacterial properties and can be applied for the further biomedical applications.

## Conclusions

In this study, we developed a simple, easy and low-cost technology for the surface modification of the 3D printing PLA disks. With the surface modification of the PLA disk by the directly absorption of antibiotic agent, we found the maximum amounts of ampicillin and vancomycin absorbed on the surface of PLA disk are around 75 and 65 mg g^−1^-PLA, respectively. With a decrease in the concentration of antibiotic agent in the aqueous solution, the amount of antibiotic agent absorbed on the sample surface also decreased. With the antibiotic agent concentration of 50 mg mL^−1^ in aqueous solution for the absorption onto the samples, the drug release profiles for the ampicillin and vancomycin from the samples in the buffer solution showed the stable drug release profiles and maintained the antibiotic agent concentration in buffer solution of higher than those for MIC 90 for the *S. aureus*. The drug release kinetics for the antibiotic agents from the samples agree well with the Korsmeyer–Peppas model. The bioactivity for the ampicillin and vancomycin with suitable amounts absorbed on the sample surface can maintain at least 28 days. The relative optical density of the *S. aureus* in the solution with the concentration 10^6^ CFU mL^−1^ can reduce into 40% using the PLA disk with directly absorbed with suitable antibiotic agents compared with that in the solution with only *S. aureus* at the same condition in test. This study shows the simple and low-cost method for the surface modification of PLA material and can have further application in the biomedical related technology.

## Conflicts of interest

There are no conflict to declare.

## Supplementary Material

RA-008-C8RA00504D-s001
