# Supplementary material for: Long-term antibacterial performances of biodegradable polylactic acid materials with direct absorption of antibiotic agents
Source: RSC Adv. 2018 May 1;8(29):16223–31. doi: 10.1039/c8ra00504d (PMC9080263; doi:10.1039/c8ra00504d)
Supplement: RA-008-C8RA00504D-s001 [file RA-008-C8RA00504D-s001.pdf]

Electronic Supplementary Material (ESI) for RSC Advances.
This journal is © The Royal Society of Chemistry 2018

Electronic Supporting Information

Long-term Antibacterial Performances of the Biodegradable Polylactic Acid Materials with Directly Absorption of Antibiotic Agents

Chien-Hao Chen[a,b], Yuan-Yuan Yao[c], Hao-Che Tang[a,b], Tung-Yi Lin[a,b], Dave W. Chen[a,b*], Kong-Wei Cheng[a,c,*]

[a] *Department of Orthopaedic Surgery, Chang Gung Memorial Hospital, Keelung Branch, Taoyuan, Taiwan*

[b] *College of Medicine, Chang Gung University, Taoyuan, Taiwan*

[c] *Department of Chemical and Materials Engineering, Chang Gung University, Taoyuan, Taiwan*

**Corresponding author*

Dave W. Chen: Address: Department of Orthopaedic Surgery, Chang Gung Memorial Hospital, Keelung Branch, Taoyuan, Taiwan

E-mail: mr5181@adm.cgmh.org.tw

K.W Cheng: Address: 259 Wen-Hwa 1st Rd., Kwei-Shan, Taoyuan 333, Taiwan

E-mail : kwcheng@mail.cgu.edu.tw

Tel.: +886-3-2118800-3353; Fax: +886-3-2118668

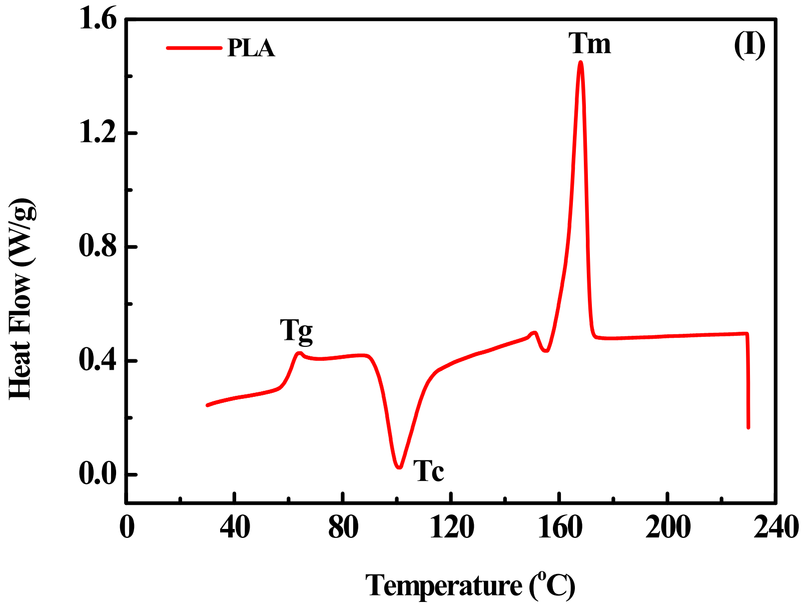
PLA
Tm
(I)
Tg
Tc
1.6
1.2
0.8
0.4
0.0
Heat Flow (W/g)
0
40
80
120
160
200
240
Temperature (°C)

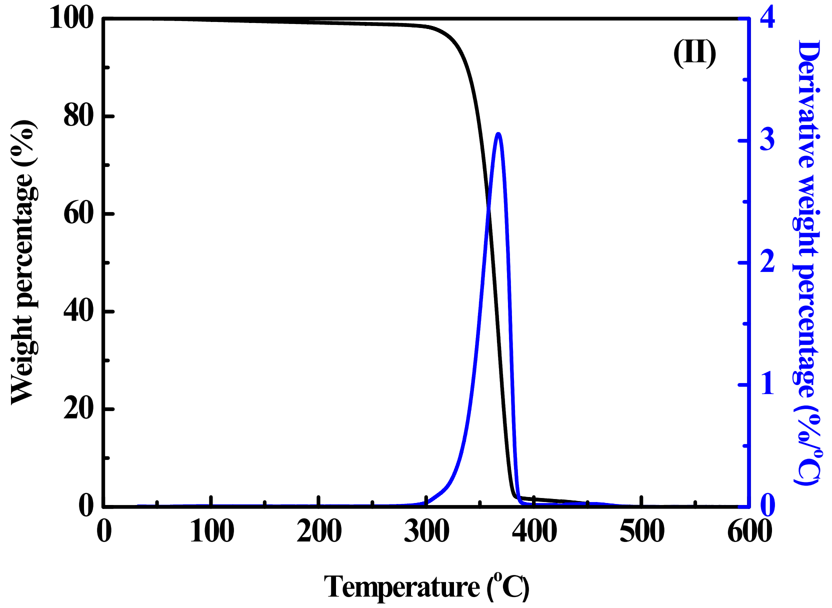
(II)
100
80
60
40
20
0
Weight percentage (%)
4
3
2
1
0
Derivative weight percentage (%/°C)
0
100
200
300
400
500
600
Temperature (°C)

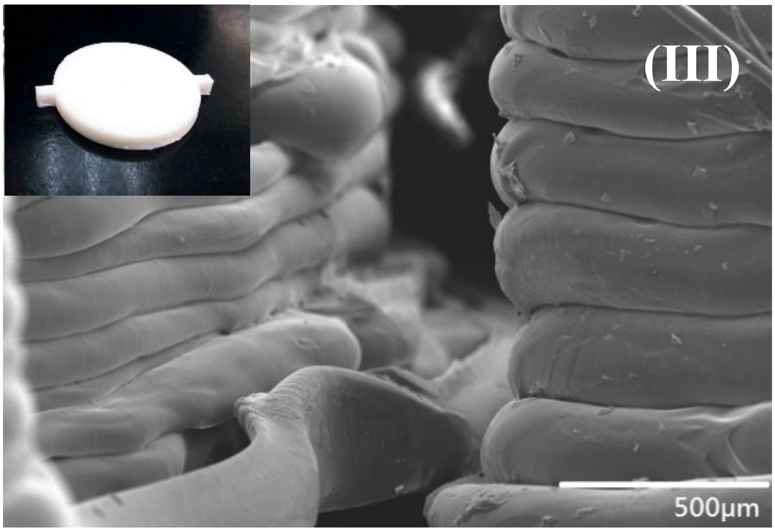

Figure S1 (I) DSC, (II) TGA and (III) SEM results of PLA materials used for the 3D printing

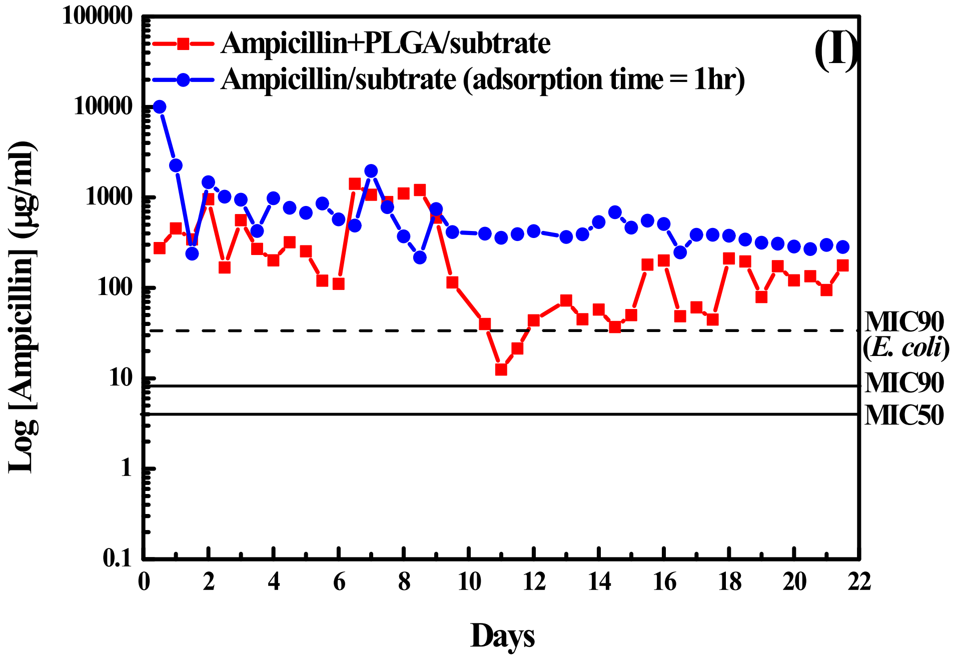

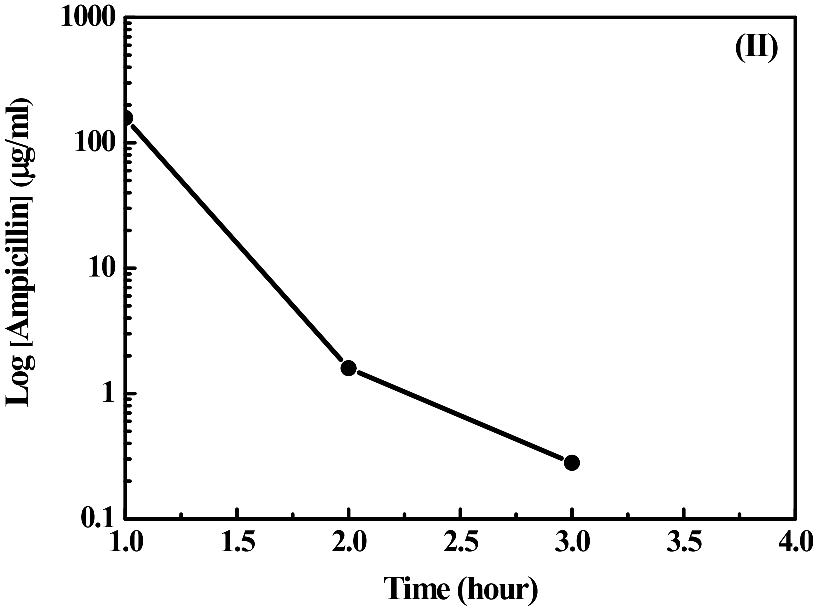

Figure S2 The release profiles of Ampicillin from (I) the PLA disk with and without the PLGA as the binder and (II) the PLA fiber in the buffer solution.

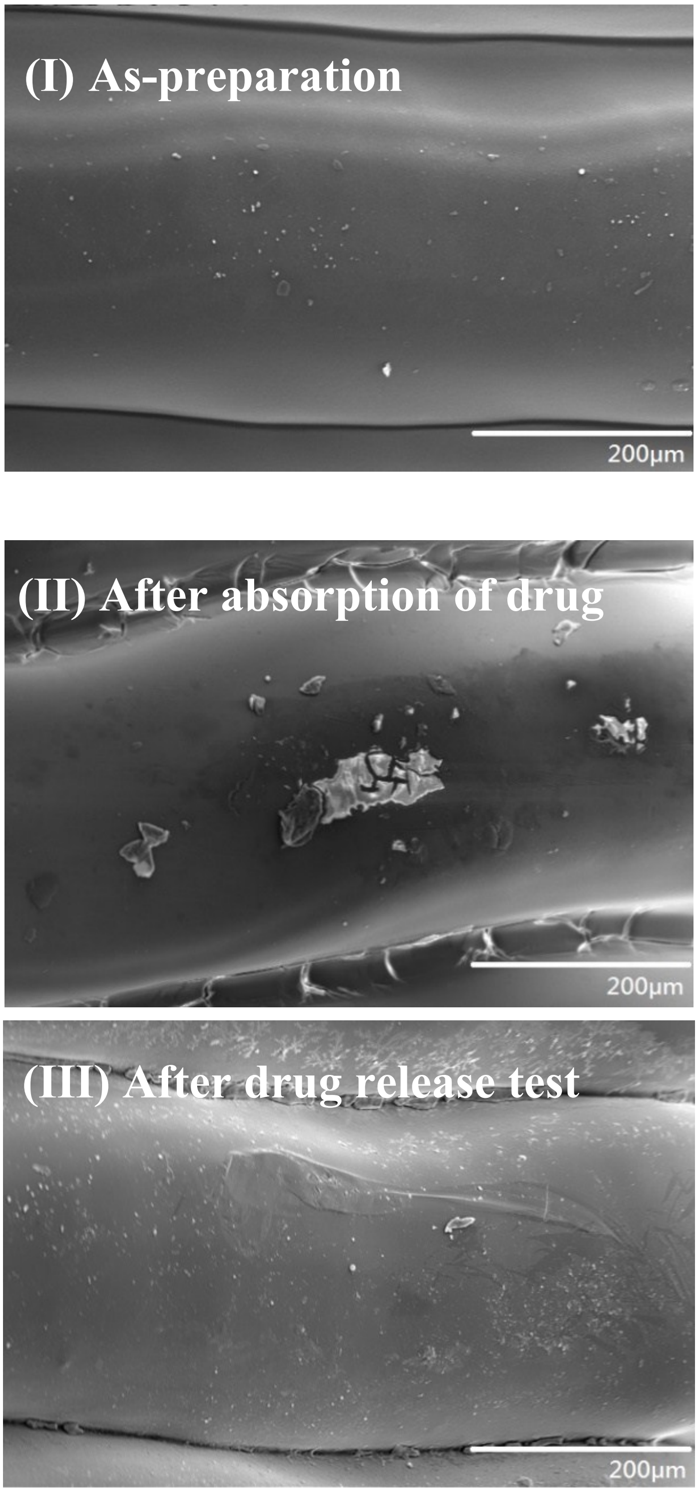

Figure S3 SEM images of the as preparation, after absorption of drug, and after drug release test for samples.

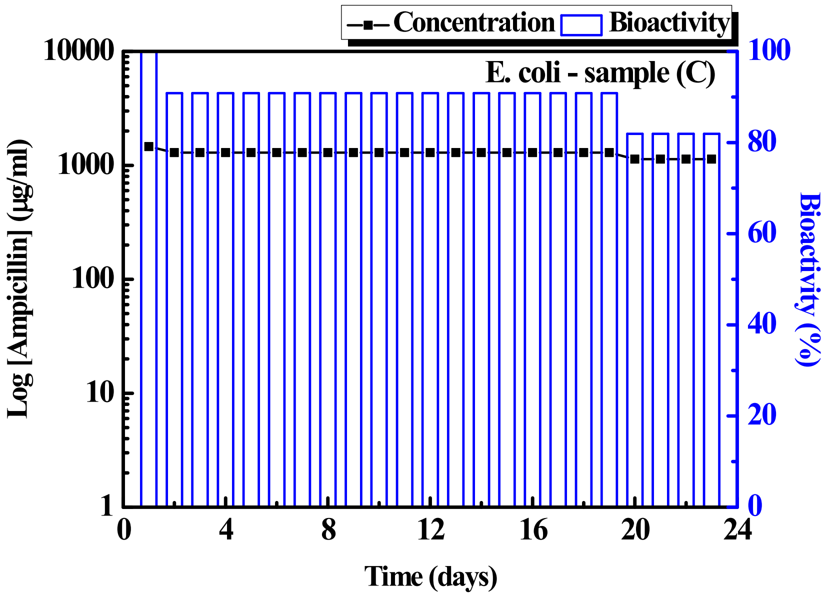

Figure S4 Bioactivity for E. coli using the release of Ampicillin from sample (C).
